# Can a Portable Flash Visual Evoked Potential (VEP) Device Identify Chiasmal Decussation Anomalies in Albinism?

**DOI:** 10.3390/diagnostics15111395

**Published:** 2025-05-30

**Authors:** Eloise Keeling, Perry Carter, Abdi Malik Musa, Fatima Shawkat, Helena Lee, Jay E. Self

**Affiliations:** 1 Clinical and Experimental Sciences, Faculty of Medicine, University of Southampton, Southampton SO16 6YD, UK; 2 Southampton Eye Unit, University Hospital Southampton NHS Foundation Trust, Southampton SO16 6YD, UK; 3 Human Development and Health, Faculty of Medicine, University of Southampton, Southampton SO16 6YDJ, UK

**Keywords:** albinism, VEP, chiasmal misrouting, OCA, decussation defects

## Abstract

**Background:** Visual evoked potentials (VEPs) are used to detect chiasmal misrouting associated with albinism. However, VEPs are only performed in specialist centres and typically have long waiting lists. The portable electrophysiology device RETeval^®^ shows promise as a clinical screening tool across a range of ophthalmic conditions. Here, we explore its utility in detecting chiasmal abnormalities associated with albinism. **Methods:** Flash VEPs were recorded on the RETeval^®^ and by standard ISCEV techniques for 27 patients with suspected albinism and 40 control patients as part of routine appointments. We retrospectively investigated the agreeability between the two methods. The amplitude/latency of the main component was measured for standard VEPs whilst a correlation value of interhemispheric difference was calculated for the RETeval^®^ data. **Results:** We demonstrate a significant difference between albinism patients and controls (*p* < 0.001) with respect to the interhemispheric difference identified by the RETeval^®^. By applying a threshold of 0.001865 to the correlation value, the RETeval^®^ detected chiasmal misrouting in all 27 patients with albinism and had 97% agreeability to standard testing. **Conclusions:** This study shows the potential of using the RETeval^®^ as a clinical tool for the diagnosis of chiasmal anomalies in albinism. The RETeval^®^ has significant time/cost savings which could hasten diagnoses.

## 1. Introduction

Oculocutaneous Albinism (OCA) is a group of inherited disorders of the melanin biosynthesis pathway, leading to hypopigmentation of the hair, skin, and eyes. OCA affects approximately 1:17,000 people worldwide [1]; however, in some populations this figure is 1:1000 [2,3]. There are seven subtypes of OCA with different genes associated with each. OCA1 is the most common form, accounting for almost 50% of cases worldwide, and is associated with mutations in the Tyrosinase gene [4]. Mutations also cause a variety of ocular features including foveal hypoplasia (as measured by OCT [5]), iris transillumination, nystagmus, excess nerve decussation at the optic chiasm, and reduced vision, often leading to patients being registered as sight impaired (Certificate of Visual Impairment; CVI). However, these features are variable and hypomorphic (mild) albinism cases are common [6,7], making accurate and reliable phenotyping, including the use of VEPs, key to diagnosis in many. Foveal hypoplasia refers to an underdevelopment of the fovea which results in a lack of the foveal pit and the persistence of inner retinal layers in the central retina. It often results in poor visual acuity and nystagmus. The most common cause of foveal hypoplasia is Albinism although other conditions can also cause it, including Aniridia (due to PAX6 mutations) and Waardenburg syndrome in which foveal hypoplasia occurs without optic nerve misrouting, as well as Foveal hypoplasia, optic nerve decussation defects, and anterior segment dysgenesis (FHONDA) syndrome in which foveal hypoplasia and optic nerve misrouting both occur. Therefore, accurate diagnosis is needed to distinguish between these conditions.

Currently, there are no treatment options available for OCA; however, recent work has shown that targeting treatments to the early postnatal development period has the potential to improve retinal morphology and function [8], and treatments targeting this period are currently being developed [9,10]. Given that this early window is limited, treatment relies on fast and accurate detection techniques to ensure that patients are diagnosed in sufficient time to allow for treatments to be given. Diagnoses can sometimes be made from genetic screening; however, as there is a high level of missing heritability in OCA1 and since it is often caused by complex genotypes [11,12,13], detailed phenotyping is needed to both raise the initial suspicion and confirm diagnosis. One such test is the visual evoked potential (VEP) used to assess optic nerve routing across the chiasm.

**Figure 1:**
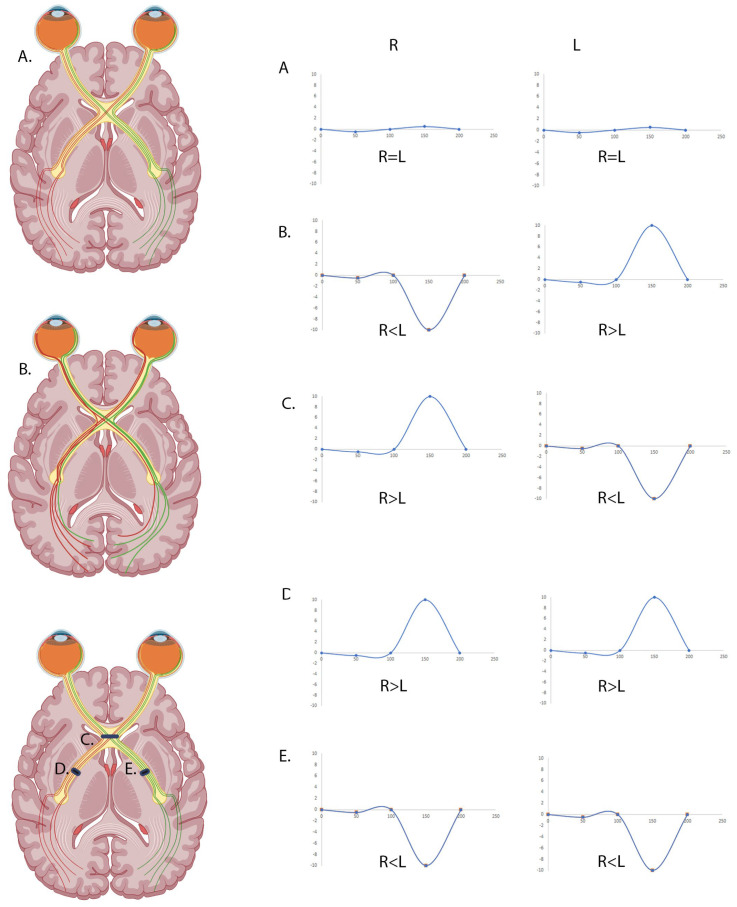
Flash VEP waveforms for normal, chiasmal, and post-chiasmal defects. Schematic sketch of predicted single electrode waveforms for monocular flash VEP. (**A**) Normal crossing waveforms for right eye (**left panel**) and left eye (**right panel**) are close to 0 at P2. (**B**) Enhanced crossing, as would be seen in albinism, causes a positive interhemispheric difference when stimulating the left eye, and a negative difference when stimulating the right eye. (**C**) A chiasmal compression lesion causes a waveform opposite to that seen in albinism where there is a negative interhemispheric difference when stimulating the left eye and a positive difference when stimulating the right eye. (**D**) A left post-chiasmal defect causes positive interhemispheric differences when stimulating both the left and the right eye. (**E**) A right post-chiasmal defect causes negative interhemispheric differences when stimulating both the left and the right eye.

VEPs are a non-invasive measure of the electrophysiological response of the brain to visual stimuli, used to monitor the function of the visual pathway from the eye, through the optic nerve, chiasm, and tract, to the visual cortex. They can help to detect various visual pathway defects, through alterations to the waveform, including albinism [6], optic neuritis [14], and compressive lesions [15] (Figure 1). VEPs are a well-established technique with recognised standards published by the International Society for Clinical Electrophysiology of Vision (ISCEV) [16] and are used worldwide to aid ophthalmic diagnosis and monitor disease progression. Less stringent protocols are employed by many expert centres to take account for less compliant patients or children such as the ‘GOSH’ paediatric protocol [17,18,19]. There are several VEP stimuli, of which pattern reversal and onset are the most common due to their qualities of lower variability and higher sensitivity, whilst flash VEPs are used in young patients and those with poor fixation/cooperation [6,20,21,22,23,24,25]. Given that diagnoses need to be made prior to the end of the postnatal development period to allow for treatment, most patients with albinism need to have VEPs performed prior to 1 year of age, meaning that the flash VEP is likely to be the appropriate test. Nonetheless, it remains difficult to perform VEPs using any technique for young children and in some cases anaesthesia is used to aid testing [26].

The drawbacks of conventional VEP systems are the requirements for expensive electrodiagnostic equipment housed in a laboratory which is not easily transported. The tests are time-consuming and require trained clinical specialists to complete and interpret, meaning that they are costly to provide. The tests are also only performed at specialist centres, resulting in long wait times and additional costs. This often prolongs the time to diagnosis and hampers the chances of meeting the identified treatment window for emerging treatment options in OCA1. Recent developments in technology have attempted to overcome some of these limitations by developing handheld portable devices capable of performing a range of ISCEV procedures [27,28]. Most of these are only suitable for adults [27], although the RETeval^®^ system (LKC, Gaithersburg, MD, USA) is also suitable for children. The RETeval^®^ also has the benefit of a step-by-step user-friendly interface and thus offers potential as a screening tool for optometrists, clinicians, or nurses to use in clinic to identify children who need further investigation or additional visual electrophysiological testing. Thus far, all studies have used the electroretinogram (ERG) function of the device, showing promising assessment of general retinal function in adults and children [28,29]. This study seeks to add to the body of work exploring the utility of the RETeval^®^ as a clinical screening tool by evaluating the potential for the flash VEP function to detect chiasmal misrouting in albinism.

## 2. Materials and Methods

This study was performed using anonymised data from a clinical service review registered with the University Hospital Southampton Health Service Review committee. As confidential patient information was not provided to anyone outside of the clinical core team and all data were anonymised to the research team, explicit consent for data from each patient was not required according to the “consent to research” guidelines as outlined by the NHS Health Research Authority in 2018.

### 2.1. Participants

In this study, all patients attending the visual electrodiagnostic department at Southampton Eye Unit had the RETeval^®^ performed as part of their routine ophthalmic evaluation. Following their appointment, anonymised data were extracted and the researcher was masked to their ophthalmic history. In total, 40 normal controls, who were found to have no anomalies on all standard ISCEV procedures, were included (aged 0–68, mean = 21; breakdown in Table 1) and were compared to 27 patients found to have chiasmal misrouting on standard VEPs (aged 0–60, mean = 7; breakdown of ages shown in Table 1 and Table 2). The age groups are defined as 0–7 and 8+ as previous reports have suggested flash VEPs to be less effective in those over 8 [6,23]. Final albinism diagnoses were given following full genotyping and phenotyping by a consultant in the Eye Unit (full details for each patient are shown in Table 2). The missing clinical details are not unexpected in the age ranges we have included, and missing heritability is a known problem in albinism genomic diagnostics; however, this final diagnosis is not critical to the research per se, as the agreeability between the two systems in detecting decussation defects is the primary research question.

### 2.2. Electrode Placement

VEPs were recorded from Ag/AgCl electrodes placed midway between OZ (10–20 system) and either ear following the paediatric Great Ormond Street Hospital montage [21]. The active electrode was placed on the right occiput, whilst the reference electrode was placed on the left occiput. A further electrode was placed at Fz as the grounding electrode.

### 2.3. VEP Recordings Using Standard Laboratory-Based EDT Techniques

Depending on the age of the patient, ISCEV-approved flash VEPs or pattern onset VEPs were performed to detect misrouting. Pattern VEPs were recorded using a black and white checkerboard pattern with a mean luminance level of 50 cd/m^2^ at 90% contrast. Patients were positioned 1 m away from the stimuli, which were presented at a frequency of 2 Hz. Initially, a check size of 1° was used, although this increased for those with poor visual acuity.

Flash VEPs were recorded using a handheld Grass photic stimulator positioned at approximately 25 cm from the patient’s eyes. Flash VEPs were presented in a dimly lit room and recorded to a brief (<5 ms) 1 Hz flash at 3 cd/s/m^2^ subtending to a visual field of at least 20° and the amplitude and latency of the major positive peak was analysed. In all scenarios, monocular VEPs were recorded and the presence or absence of chiasmal misrouting detected by comparing the monocular VEP trans-occipital distribution. Flash stimulation of the left eye typically elicits a negative VEP component recorded over the right occipital cortex, whereas stimulation of the right eye elicits a negative component over the left occiput. If a trans-occipital asymmetry of the VEP from one eye is mirrored across the midline by the other eye, it is termed a ‘crossed asymmetry’, or abnormal crossing.

### 2.4. VEP Recordings Using the RETeval^®^

**Figure 2:**
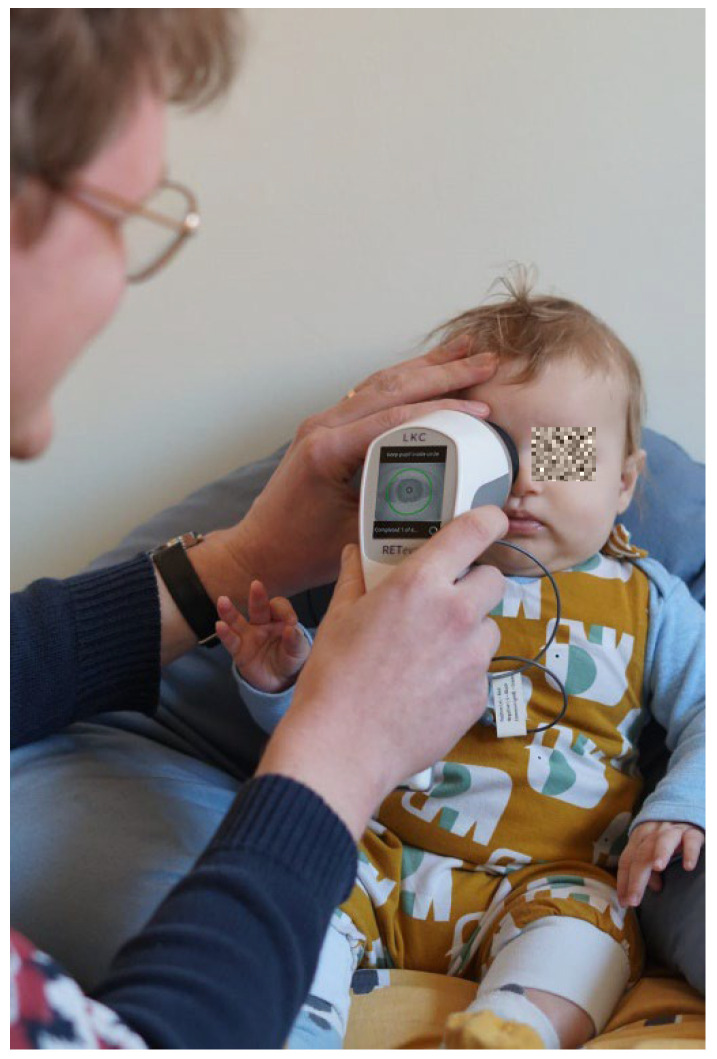
Example RETeval^®^ set up on a baby. The permission was obtained from the parent to use the photo.

The handheld RETeval^®^ system (LKC, USA) can perform ISCEV-approved protocols [16] in a user-friendly manner (Figure 2). A flash of 3 cd·s/m^2^ was recorded with a visual field of at least 20°. An average was taken from 10 brief (<5 ms) 1 Hz flashes per recording, and recordings were repeated at least twice per eye. Each eye was tested separately and the VEP waveform recorded by the left electrode was automatically subtracted from that recorded by the right electrode to create a single channel flash VEP output known as the interhemispheric difference.

### 2.5. Analysis

For all patients a ‘RETeval^®^ score’ was calculated by performing Pearson’s correlation analysis using GraphPad Prism 10 to quantify the difference in interhemispheric differences (the single channel flash VEP) between the 2 eyes. Only recordings between 0 and 200 ms were used as this is the window with the most significant asymmetry for younger children [25]. A RETeval^®^ score of +1.0 shows complete correlation whereas a value of −1.0 shows complete asymmetry. The more negative the score the more abnormal the crossing is, and any score less than 0 was termed abnormal. Unpaired *t*-tests were then performed to compare the RETeval^®^ score of the control data with the albinism data. Receiver Operating Characteristic (ROC) curves were generated in GraphPad Prism to calculate the sensitivity and specificity of the device. Data were also grouped by age and differences across age groups for control and chiasmal misrouting data were measured by a 2-way ANOVA in GraphPad Prism.

## 3. Results

### 3.1. Agreeability Between Standard Clinic VEPs and RETeval^®^ VEP in Detecting Chiasmal Pathway Anomalies

**Figure 3:**
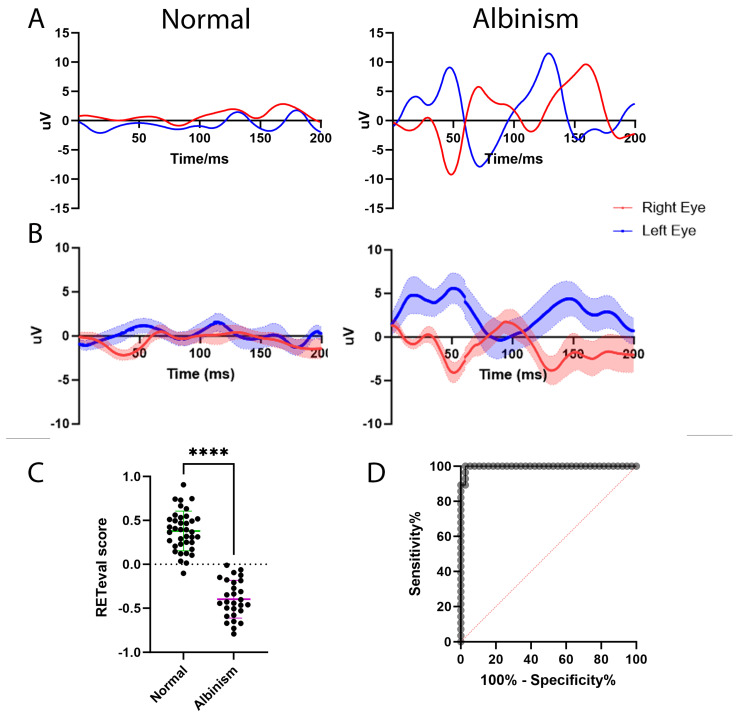
Using the RETeval^®^ to detect misrouting across the chiasm as seen in albinism. (**A**) Representative waveforms of recordings using the RETeval^®^ for control patients (**left panel**) and albinism patients (**right panel**). (**B**) Grouped averages for control and albinism patients were generated where the main line is the mean and the shaded area shows SD. (**C**) Comparison of the RETeval^®^ score for control and albinism patients. The RETeval^®^ score was generated by calculating a Pearson’s correlation value for each patient to compare the interhemispheric differences from the left eye and the right eye. An unpaired *t*-test was performed to calculate the statistical significance between the RETeval^®^ scores for each group (**** shows *p* < 0.0001). (**D**) An ROC curve for detecting chiasmal crossing anomalies using the RETeval^®^. The red line represents the ROC curve for a random guess whilst the grey line is the curve for our data. The area under the curve is 0.9972, with a confidence interval of 0.9903 to 1.000 showing the device to be highly sensitive and specific in detecting misrouting across the optic chiasm. Using the ROC curve, a cut-off value of 0.001865 gives 100% sensitivity and 97% specificity.

Forty healthy controls and twenty-seven patients with chiasmal misrouting were included in this study (Table 2). All patients were selected from those undergoing routine EDTs (VEP and ERG) in a regional referral unit. Alongside standard ISCEV protocol EDTs, these patients also had flash VEP recordings measured using the handheld RETeval^®^ as part of their routine clinical testing and data were then anonymised and extracted for use in this study (Figure 3A). All but 1 of the 27 albinism patients showed abnormal crossing by standard testing whilst all 27 had abnormal VEPs recorded by the RETeval^®^. Similarly, all but 1 of the 40 controls that had normal standard VEP results were also normal on the RETeval^®^. Together, this showed 97% agreement to standard ISCEV protocols. Grouped averages were created for both groups (Figure 3B) which showed almost flat interhemispheric differences in the healthy patients, and a peak in the interhemispheric difference from the right eye of the albinism patients at 100 ms and a trough at the same latency and with the same amplitude in the interhemispheric difference from the left eye.

### 3.2. Analysing the Full Epoch May Increase the Sensitivity of the RETeval^®^ VEP to Identify Chiasmal Misrouting

Pearson’s correlation analysis was performed to mathematically analyse the epoch, creating a RETeval^®^ score for each patient. As detailed above, all but one of the healthy controls had a RETeval^®^ score of 0 or above. Patients with no noise in the traces and perfectly symmetrical crossing should have a score of exactly 0; however, in reality there is a slight positive correlation between the two channels. This can be explained due to brains not being exactly symmetrical in the skull, and therefore one electrode will detect a larger signal regardless of the eye being stimulated, thus making the RETeval^®^ score trend in a positive direction. The single control patient who had a negative RETeval^®^ score showed a weak negative correlation with a score of −0.10180. The albinism patients all had negative RETeval^®^ scores, as would be expected when there is an asymmetry across the chiasm; however, one patient did not show asymmetry on standard testing. Data were normally distributed and therefore an unpaired *t*-test was performed to compare the RETeval^®^ score of the control data (mean = 0.3697) with that of the chiasmal misrouting data (mean = −0.3979) and this showed strong significance (*p* < 0.0001). We calculated a correlation score for the singular albinism patient who had a negative correlation detected on the RETeval^®^ but no chiasmal misrouting when looking at the amplitude and latency of the main component by standard testing. Interestingly, by looking at the entire epoch in this way, a misrouting was correctly detected (correlation of −0.5650) which was not identified when just looking at the main component, increasing the agreeability of the two systems to 98.5%.

### 3.3. The RETeval^®^ Is Highly Sensitive and Specific at Detecting Chiasmal Misrouting

An ROC curve was created to estimate the sensitivity and specificity of the RETeval^®^ in detecting chiasmal misrouting (Figure 3C). The area under the curve was found to be 0.9972 and had a confidence interval of 0.9903 to 1.000. From this data, a cut-off point of 0.001865 would give 100% sensitivity and 97% specificity (0% false negatives and 3% false positives). When applying these cut-off points to all patient data collected by the RETeval^®^, 65 out of all 67 patients included in the study would have received the same results as by the standard VEPs, and 2 would have had false positives. However, one of the false positives still would have gone on to receive a chiasmal misrouting diagnosis and misrouting was also found by standard testing when the entire epoch was analysed, suggesting that it may not actually be a false positive and in fact we only had one false positive.

### 3.4. The RETeval^®^ Score Is Not Age Dependent

**Figure 4:**
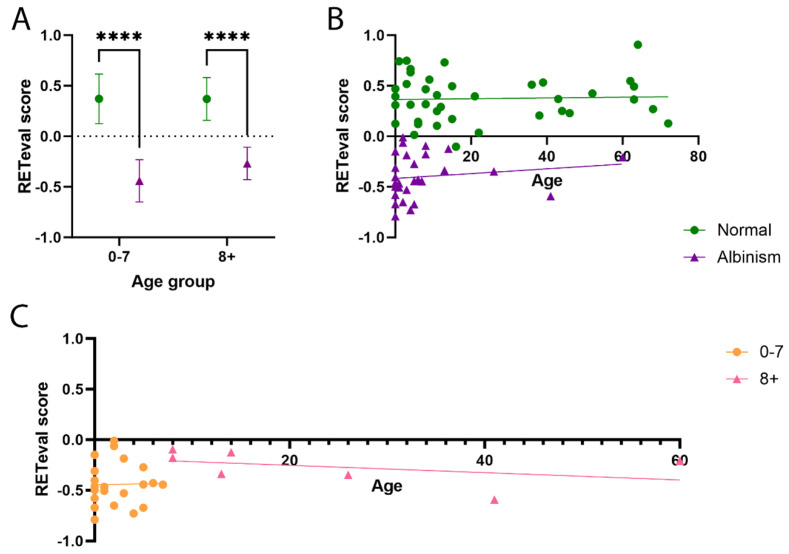
Using the RETeval^®^ to detect misrouting is not age dependent. (**A**) Comparison of the RETeval^®^ score for controls and albinism across the age groups, with mean and SD shown for each group. Two-way ANOVAs were performed using GraphPad Prism (**** denotes *p* < 0.0001). (**B**) Correlation of the RETeval score with age for controls and albinism patients. Linear regression analysis was performed and it found that age did not affect the RETeval^®^ score for either group (controls: R^2^ = 0.001501 *p* = 0.8148; albinism: R^2^ = 0.0223, *p* = 0.4482). (**C**) Correlation of the RETeval^®^ score with age for the albinism patients split by age group. Again, linear regression analysis showed that age did not affect the RETeval^®^ score for either age group (0–7: R^2^ = 0.0003628, *p* = 0.9347; 8+: R^2^ = 0.1697, *p* = 0.3584).

The RETeval^®^ scores were plotted against the age of the patients to determine the role age has on the potential use of the RETeval^®^ device (Figure 4). An R^2^ value of 0.001501 (*p* = 0.8148) was found for the control group, showing that the RETeval^®^ score for normal crossing is unaffected by age. For the chiasmal misrouting group, there was a slight positive trend (R^2^ = 0.0223; *p* = 0.4483) however this was not significant (Figure 4A,B). When plotting the scores for the two age groups (0–7 and 8+), the slight positive trend was removed in the 0–7 group (R^2^ = 0.000362; *p* = 0.9347) (Figure 4C). Taken together, these results show that age does not affect the use of the RETeval^®^ in detecting misrouting across the chiasm associated with albinism and analysing the entire epoch rather than just looking at the amplitude and latencies of the main component(s).

## 4. Discussion

Recent studies have shown the potential of using the RETeval^®^ as a point-of-care triaging tool [28,29]. The aim of this study was to investigate the use of the RETeval^®^ in detecting chiasmal misrouting associated with albinism to reduce the time to diagnosis.

Since there is a short window for treatment [8], streamlining diagnosis is of considerable benefit to the patient.

Using correlation coefficients to detect misrouting is a known technique that has struggled to gain traction in a real-world clinical setting. Previous studies have used both Pearson’s correlation and chiasm coefficient to compare interhemispheric difference from right eye and left eye, the latter including a high-pass filter to cope with drift [22,30]. We have chosen not to perform a chiasm coefficient due to the high sensitivity and specificity in our data. In addition, since we are suggesting the RETeval^®^ predominantly as a point-of-care tool, there is a need to minimise post-testing data analysis to keep it as user-friendly as possible. In theory, a person with normal crossing would have a Pearson’s correlation score close to 0; however, in reality a person with normal crossing often has a correlation score of between 0 and 1 due to occipital petalia [31]. In our study, the Pearson’s correlation data from control patients were localised between 0 and +1, showing the ability of the RETeval^®^ to identify normal chiasmal decussation. Interestingly, the range of data was higher than anticipated which could be due to variations in brain asymmetries giving an overall positive correlation. This is common in other studies using correlation coefficients [24].

In all cases of suspected albinism included in this proof-of-concept study, the RETeval^®^ was able to detect anomalous crossing at the optic chiasm, with high significance, when compared with controls. No patients with misrouting detected using standard procedures had normal crossing on the RETeval^®^; however, the RETeval did detect misrouting in a patient that was not detected by standard ISCEV protocols. Interestingly, when analysing the full epoch taken via standard testing rather than just the main components, a misrouting was detected and this patient did go on to receive an albinism diagnosis following genotyping, highlighting the need to use multiple testing methods even in patients > 8 years old, and not just relying on measuring the amplitude and latency in pattern onset VEPs. Unfortunately, since the RETeval^®^ automatically calculates the interhemispheric difference and given the numerous methods that can detect misrouting following standard ISCEV procedures (e.g., pattern onset and half-field VEPs), it is not possible to directly compare the two systems, other than the overall agreeability of diagnosis. This was found to be 97%, which increased to 98.5% when the entire epoch was analysed for the ISCEV procedure VEPs, rather than just analysis of the main components.

The sensitivity and specificity outcomes for the RETeval^®^ detecting misrouting associated with albinism can be considered outstanding (>0.9 AUC) [32] with a strong confidence interval. Previous studies have suggested that the highest specificity possible could be around 85% [22,25]; however, we have shown a much higher specificity than this. This high specificity could be attributed to a selection bias as we included subjects from nystagmus clinics, and therefore we may have excluded some hypomorphic cases which, by definition, could be the patients with no (or harder to detect) misrouting. The high specificity could also be due to the RETeval^®^ requiring less patient cooperation than other recording types, and other handheld flash VEP systems have also shown higher specificities when compared with standard flash VEPs [22]. This study proves the potential of generating a cut-off point that could be used when using the RETeval^®^ as a screening tool. Using the data generated here, a cut-off value of 0.001865 would give zero false negatives, and therefore no-one who was found to have normal crossing on the RETeval^®^ would go on to have abnormal crossings on the ISCEV, and no albinism patients would be excluded from going on to further studies. However, more in-depth analysis of a much larger patient set would be required before this cut-off value could be used in a clinical setting.

Despite numerous reports previously showing flash VEP to be less effective in patients aged over 7 [6,23], this is not corroborated in our data. One reason for this could be that previous reports have followed ISCEV procedures of looking at the amplitude and latency of the main component. Due to the hardware limitations of the RETeval^®^, we were unable to analyse the data in this standard way and instead looked at the entire epoch and calculated the correlation of the interhemispheric difference for both eyes. As previously mentioned, this technique has been performed before but on smaller sample sizes [24,25,30] and has struggled to gain traction in a clinical setting. This could be due to it requiring relatively complex post-analysis mathematics which have not been incorporated into systems by the manufacturers. This study could therefore help to distinguish Pearson’s correlation or chiasmal coefficient as a way of using flash VEP to identify chiasmal misrouting as a standard ISCEV procedure in the future by overcoming some of these previous limitations. Indeed, the albinism patient who did not have misrouting recorded by looking at the main component on standard VEPs but did when the correlation across the entire epoch was calculated using both the RETeval^®^ and standard VEP data was >8 years of age. This highlights the need for larger, in-depth studies to be performed using ISCEV flash VEP protocols for comparison when looking at the full epoch rather than the amplitude and latencies of the main component.

Since this study is a retrospective service review, there are several limitations to this work that need to be corrected before the RETeval^®^ can be adopted in a real-world setting, In this study, a relatively small sample size was included from one tertiary centre, which could exaggerate sensitivity and specificity, therefore a multi-centre study testing for use in paediatric patients in hypomorphic albinism cases needs to be completed to fully compare the RETeval^®^ and standard testing techniques, and to generate an unbiased cut-off value.

In the future, the RETeval^®^ could potentially be used to detect other post-chiasmal abnormalities, such as hemispheric lesions or chiasmal compression tumours (as shown in Figure 1). In such instances, chiasmal compressive lesions could also be detected using RETeval^®^ score calculations but retro-chiasmal anomalies would only be identifiable upon visual analysis of the waveforms and could not be detected using Pearson’s correlation. In such patients, both occiputs receive the same positive or negative input and calculating the difference across the hemisphere would give a score between 0 and 1, as would be seen in normal controls. For similar reasons, the device is unable to look at pre-chiasmal abnormalities using this method as, if the dysfunction is prior to the optic tract, signals on both sides of the head would be affected and, as a result, both electrodes would receive the same input. This highlights the importance of the user understanding the potential defects and selecting the correct test and analysis to use as, if the wrong test is selected, abnormalities could easily be missed.

## 5. Conclusions

In conclusion, this study demonstrates that the RETeval^®^ system shows potential as a point-of-care triaging/screening tool for non-expert clinicians to help provide diagnostic information and streamline patient management in cases of suspected albinism. This will help to minimise time to diagnosis and enable patients to have the maximum time possible inside the limited therapeutic window. Importantly, the sensitivity of the technique was very high (suggesting a low risk of false negative findings), and the specificity was also high with only a small percentage of false positive findings. This would be ideal for a screening tool. Further studies are required to refine the parameters of using the device in this scenario and such studies could potentially also investigate chiasmal and retro-chiasmal pathologies. It is important to note that the RETeval^®^ device would not and cannot replace full visual electrodiagnostic assessment where other tests such as pattern VEPs, ERGs, and electro-oculograms may be necessary to build a complete clinical picture. Instead, it might offer potential as an additional clinical tool which could be used by relatively untrained clinical staff as a screening device. Further work is needed to determine its acceptability to patients, define its useability in non-expert hands, and to calculate the thresholds for use as a screening tool to detect decussation defects in albinism.

## Figures and Tables

**Table 1:** Ages and number of albinism participants included in the study.

	Control	Albinism
0–7	15	20
8+	25	7

**Table 2:** Phenotypes and genotypes of patients with chiasmal misrouting included in the study.

Age (Years)	Iris Transillumination	Nystagmus	Pale Fundus	Foveal Hypoplasia	RETeval Pearson‘s Correlate	Genomics Variant 1	Genomics Variant 2	Genomic Diagnostic Summary	Albinism Diagnosis
5	No	No	Yes	Yes (Grade 3)	−0.6725	TYR c.1217C>T p. (Pro406Leu)	TYR c.1036G>A p. (Gly346Arg)	OCA1	proven
1	Yes	Yes	Yes	Yes (Grade 1/2)	−0.465	OCA2 c.1327G>A, P.(Val443lle)	nil	Likely OCA2 with missing variant	likely
0	Yes	Yes	Yes	Yes (Grade 1/2)	−0.3107	nil	nil	Clinical OCA but no variants on R39 panel	likely
3	Yes	Yes	Yes	No	−0.5299	nil	Hom R402Q and Het S192Y	Likely OCA1b but segregation not complete	likely
9	No	No	Yes	Yes (Grade 3/4)	−0.09339	TYR c.823G>T p.(Val275Phe)	het s192y and het r402Q	OCA1b	proven
1	Yes	Yes	Yes	Yes (Grade 3)	−0.4979	nil	nil	Clinical OCA but no variants on R39 panel	likely
1	No	Yes	Yes	Yes	−0.5061	nil	nil	Clinical OCA but no variants on R39 panel	likely
1	No	Yes	Yes	Yes	−0.6705	OCA2 c.1255C>T p.(Arg419Trp)	nil	Likely OCA2 with missing variant	likely
0	Unknown	Yes	Yes	Yes	−0.7914	nil	nil	Clinical OCA but no variants on R39 panel	likely
7	Yes	Yes	Yes	Yes (Grade 4)	−0.4442	TYR c.229C>T p.(Arg77Trp)	TYR s192Y (hom) and R402Q (het)	Likely OCA1b but segregation not complete	likely
1	No	Yes	Yes	Yes (Grade 1/2)	−0.4607	nil	nil	Clinical OCA but no variants on R39 panel	likely
5	Yes	Yes	Yes	Unknown	−0.7293	Unknown	Unknown	Likely OCA1b but segregation not complete	likely
4	Yes	No	Yes	Yes	−0.1855	TYR c.650G>A p.(Arg217Gln)	TYR s192Y (het) and R402Q (hom)	Likely OCA1b but segregation not complete	likely
1	Yes	No	Yes	Unknown	−0.4032	nil	nil	Not done	possible
2	Yes	Yes	Unknown	Unknown	−0.01125	nil	nil	Not done	possible
26	Yes	Yes	Yes	Yes (Grade 3/4)	−0.3472	TYR c.1118C>A p.(Thr373Lys)	TYR s192Y (hom) and R402Q (het)	OCA1b	proven
60	No	Yes	No	Yes (Grade 1)	−0.2073	TYR c.1118C>A p.(Thr373Lys)	TYR s192Y (het) and R402Q (het)	OCA1b	proven
2	No	No	Yes	Unknown	−0.6503	nil	nil	Not done	possible
41	No	Yes	Yes	Yes (Grade 3)	−0.5923	nil	nil	Not done	possible
5	Unknown	no	yes	Yes (Grade 3)	−0.442	nil	nil	Not done	possible
13	Yes	No	Yes	mild	−0.3373	OCA2 c.1025A>G p. (Tyr342Cys)	OCA2 c.1418T>A p.(lle473Asn)	OCA2	proven
14	Yes	Yes	Yes	Yes	−0.1239	OCA2 c.1025A>G p. (Tyr342Cys)	OCA2 c.1418T>A p.(lle473Asn)	OCA2	proven
0	Yes	No	Yes	Yes (Grade 1)	−0.5789	nil	nil	Clinical OCA but no variants on R39 panel	likely
6	Unknown	no	Yes	Yes Grade 3	−0.4285	TYR c.1118C>A p.(Thr373Lys)	nil	Likely OCA1 with missing variant	likely
5	Yes	Yes	yes	Yes Grade 3	−0.2729	OCA2 c.619_636del p.(Leu207_Leu212del)	OCA2 c.1103C>T p.(Ala368Val)	OCA2	proven
2	Yes	Yes	Yes	Unknown	−0.06207	nil	nil	Clinical OCA but no variants on R39 panel	likely
0	Unknown	Yes	Unknown	Unknown	−0.1491	nil	nil	Not done	possible

## Data Availability

The raw data supporting the conclusions of this article will be made available by the authors on request.

## Abbreviations

The following abbreviations are used in this manuscript:

VEP	Visual Evoke Potentials
ISCEV	International Society for Clinical Electrophysiology of Vision
OCA	Oculocutaneous Albinism
